# New Insights Into the Therapeutic Management of Varicella Zoster Virus Meningitis: A Series of 123 Polymerase Chain Reaction–Confirmed Cases

**DOI:** 10.1093/ofid/ofae340

**Published:** 2024-06-20

**Authors:** Marie Dulin, Sylvie Chevret, Maud Salmona, Hervé Jacquier, Béatrice Bercot, Jean-Michel Molina, David Lebeaux, Anne-Lise Munier

**Affiliations:** Department of Infectious Diseases, Saint Louis-Lariboisière Hospital, Assistance Publique-Hôpitaux de Paris, Université Paris Cité, Paris, France; Biostatistics Department, Saint Louis Hospital, Assistance Publique-Hôpitaux de Paris, Université Paris Cité, Paris, France; Laboratory of Virology, Saint Louis-Lariboisière-Fernand-Widal Hospital Group, Assistance Publique-Hôpitaux de Paris, Université Paris Cité, Paris, France; Laboratory of Microbiology, Saint Louis-Lariboisière-Fernand-Widal Hospital Group, Assistance Publique-Hôpitaux de Paris, Université Paris Cité, Paris, France; Laboratory of Microbiology, Saint Louis-Lariboisière-Fernand-Widal Hospital Group, Assistance Publique-Hôpitaux de Paris, Université Paris Cité, Paris, France; Department of Infectious Diseases, Saint Louis-Lariboisière Hospital, Assistance Publique-Hôpitaux de Paris, Université Paris Cité, Paris, France; Department of Infectious Diseases, Saint Louis-Lariboisière Hospital, Assistance Publique-Hôpitaux de Paris, Université Paris Cité, Paris, France; Department of Infectious Diseases, Saint Louis-Lariboisière Hospital, Assistance Publique-Hôpitaux de Paris, Université Paris Cité, Paris, France

**Keywords:** acyclovir, aseptic meningitis, neurological complications, valacyclovir, varicella zoster virus

## Abstract

**Background:**

Varicella zoster virus (VZV) can reactivate and cause meningitis, but few studies have distinguished it from meningoencephalitis regarding treatment recommendations.

The objective of this study was to assess the outcomes of a large series of patients with VZV meningitis according to their therapeutic management.

**Methods:**

We conducted a bicentric retrospective cohort study, in Paris, France, including all adult patients with a cerebrospinal fluid sample positive for VZV by polymerase chain reaction between April 2014 and June 2022. We distinguished meningitis from encephalitis according to the International Encephalitis Consortium criteria. Unfavorable outcome was defined as mortality or functional sequelae defined by a loss of 2 points on the modified Rankin Scale.

**Results:**

We included 123 patients with meningitis. Among them, 14% received no antivirals, while 20% were treated with oral valacyclovir alone, 41% with a short course of intravenous (IV) acyclovir before switch to valacyclovir, and 25% with a long course of IV acyclovir. Outcomes were favorable regardless of antiviral regimen. In multivariate analysis, only age, underlying immunosuppression, and cranial radiculitis appear to be predictive factors for longer IV therapy, based on the Akaike information criterion.

**Conclusions:**

In this study, patients with VZV meningitis had a good outcome, with no evidence of any impact of the treatment strategy. However, further studies are needed to support the possibility of milder treatment in immunocompetent patients, avoiding cost and side effects of IV acyclovir.

## Teaser

Patients with varicella zoster virus meningitis are mostly young and immunocompetent, and their outcome is good even in the absence of antiviral treatment, suggesting that they could benefit from a milder treatment than intravenous acyclovir, avoiding costs and side effects.

Varicella zoster virus (VZV) is a double-stranded DNA alpha-herpesvirus that causes chickenpox and herpes zoster (HZ) [1]. In most temperate climates, >80% of people are infected before adolescence, making it a widespread ubiquitous virus [2]. After primary infection, it establishes latent infection in trigeminal and dorsal root ganglia for the entire life of the host [3–5]. From this reservoir it can reactivate to cause HZ and a wide variety of neurological disorders, such as meningitis, encephalitis, myelitis, radiculitis, cerebral vasculitis, or necrotizing retinitis [3, 6]. These complications may occur throughout life, with or without associated HZ rash [7–11].

In some observational studies, VZV now appears to be the most common cause of sporadic viral meningitis [8, 12, 13]. While immunosuppression and age-related decline in specific T-cell responses appear to be well-established risk factors for HZ [14], predisposing factors for neurological complications are more controversial [15]. Recent small case series have suggested that VZV meningitis preferentially affects young and immunocompetent subjects with a good prognosis [8, 11, 15–17], but few studies have distinguished it from VZV meningoencephalitis, making management recommendations difficult to interpret.

According to the Infectious Diseases Society of America (IDSA) guidelines, intravenous (IV) acyclovir at a dose of 10–15 mg/kg 3 times daily for 10–14 days is the gold standard for zoster infection of the central nervous system (CNS) [18]. However, there are no strong evidence-based data to support such a recommendation.

In case reports and case series, VZV meningitis has been successfully treated with various regimens: long course of IV acyclovir; short course of IV acyclovir followed by oral valacyclovir; and, rarely, valacyclovir alone or no antivirals [15–17, 19].

Our hypothesis was that high-dose IV acyclovir, which induces a longer hospital stay and frequent renal side effects [20, 21], might not be necessary in these patients, as described for herpes simplex (HSV) type 2 meningitis [22].

The aim of this study was to compare across treatment groups, the clinical and paraclinical features, and the outcomes of a large series of VZV meningitis, in French hospitals.

## MATERIALS AND METHODS

### Study Design

This retrospective study was conducted in 2 university hospitals in Paris, France (Lariboisière and Saint Louis hospitals), between April 2014 and June 2022. One of these hospitals is associated with a regional reference center for the management of headaches, and both have implemented a multiplex polymerase chain reaction (PCR) for the diagnosis of infectious meningitis in routine since 2017 (BioFire FilmArray ME [meningitis/encephalitis] panel from bioMérieux, Marcy l’Etoile, France) [23]. This multiplex PCR can detect 14 pathogens, including VZV, and is systematically performed for cerebrospinal fluid (CSF) with a white blood cell (WBC) count >10 cells/μL. A qualitative triplex real-time PCR (VZV, HSV-1, HSV-2) was performed at the request of physicians in cases of clinical suspicion (RealStar alpha-herpesvirus PCR kit 1.0, Altona Diagnostics, Joué-les-Tours, France).

All adult patients (≥16 years of age) with a CSF sample positive for VZV by PCR method (triplex and/or multiplex) during the study period were included. After reviewing medical records, patients without clinical signs of CNS infection were excluded.

### Definitions of CNS Infection

VZV meningitis was defined as follows: patients with WBC count in CSF ≥5 cells/μL with meningeal symptoms (unusual headache and/or neck stiffness), with or without cranial nerve involvement, but without clinical or paraclinical evidence of associated encephalitis.

VZV encephalitis was defined as follows: patients with clinical signs of encephalopathy (altered mental status, seizure, and/or focal central neurological deficit) and/or paraclinical signs of brain damage (focal abnormality on electroencephalogram [EEG] or neuroimaging) as defined by the International Encephalitis Consortium [24].

### Data Collection

The following data were extracted from electronic medical records for all included patients: demographics (age, sex, body mass index), comorbidities and immunosuppressive factors, clinical manifestations, laboratory findings (blood and CSF analysis), brain imaging by computed tomography (CT) or magnetic resonance imaging (MRI), and EEG results if performed.

Patients with meningitis were studied further with a specific look at management (empiric antibiotics, antiviral, corticosteroids), complications (acute kidney injury), and clinical outcomes (length of hospital stay, delta on the modified Rankin Scale [mRS 17, 25] between preinfection and postinfection disability status, mortality, length of follow-up).

### Study Outcomes and Follow-up

Outcome was considered unfavorable if the patient died or had a delta mRS of at least 2 points at discharge. Clinical outcomes were assessed at the end of meningitis treatment and at the end of follow-up for patients with subsequent visits to one of the Paris public assistance hospitals (AP-HP). Patients who did not return to any AP-HP hospital were considered lost to follow-up. Their survival status was verified on a French public death registry (www.deces-en-france.fr).

### Statistical Analysis

We first aimed to compare and define a set of baseline characteristics associated with the treatment groups, distinguishing 4 levels—namely, IV acyclovir for ≥7 days, IV acyclovir for <7 days (with or without oral switch), oral valacyclovir only, and no antivirals. Univariable comparisons between those groups used the exact Fisher test or the Kruskal-Wallis test. Multivariable analyses used multinomial log-linear regression models. Second, we restricted the analyses in patients who received acyclovir, to compare the characteristics of patients who received a long (≥7 days) versus a short (<7 days) treatment duration. Univariable analyses used the exact Fisher test or the Kruskal-Wallis test, while logistic models were used to quantify the strength of association by odds ratio in univariable or multivariable models. Variable selection in multivariable models used the Akaike information criterion (AIC), which aims to select the model with the highest predictive accuracy. All statistical tests were 2-sided with *P* values <.05 considered statistically significant. All data were analyzed using R 4.1.1 software (https://www.R-project.org/). The R package net was used.

### Patient Consent Statement

This study was carried out in accordance with the Declaration of Helsinki and was approved by the French ethics committee in infectious diseases research (CER-MIT 2023-0306). All patients received an information letter about the study and approved the use of their medical data.

## RESULTS

### Study Population

**Figure 1.**
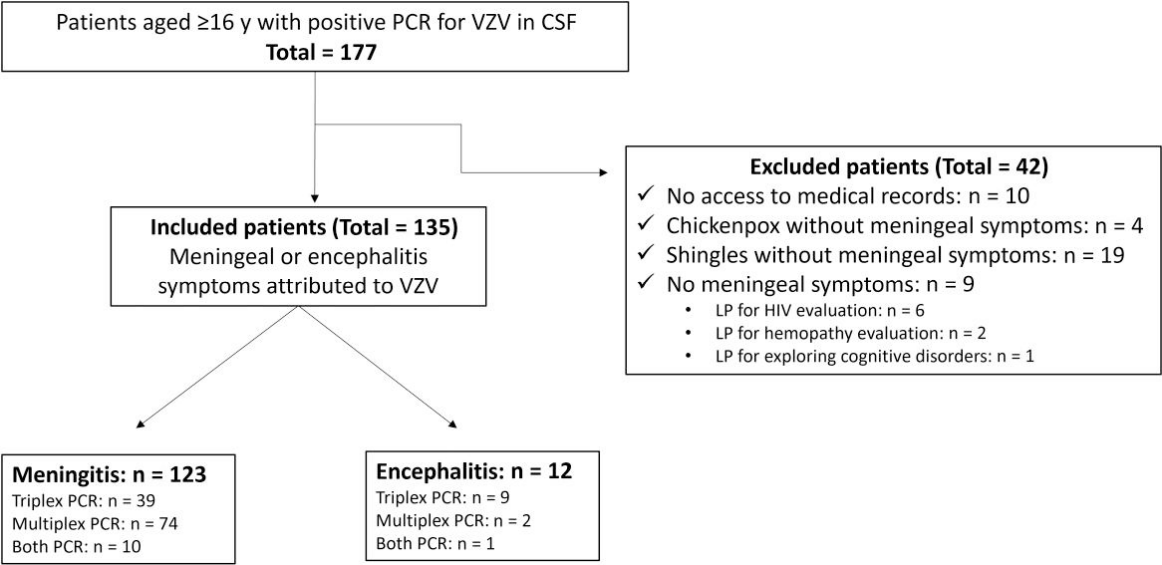
Study flowchart. Abbreviations: CSF, cerebrospinal fluid; HIV, human immunodeficiency virus; LP, lumbar puncture; PCR, polymerase chain reaction; VZV, varicella zoster virus.

Over the 8-year period, we identified 177 patients with positive VZV PCR in CSF. After excluding those who did not meet the diagnostic criteria for CNS infection (n = 32) and those whose medical records were not accessible (n = 10), we included 135 patients: 123 with meningitis and 12 with encephalitis (Figure 1).

**Figure 2.**
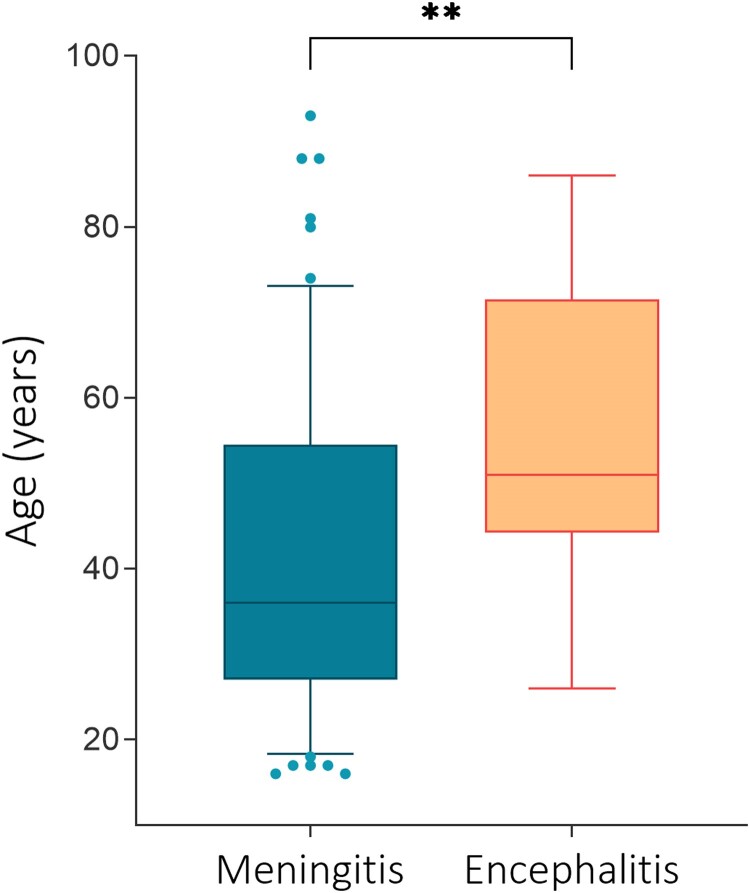
Age distribution at diagnosis of meningitis versus encephalitis. **p = 0.0048.

Demographic and clinical features of included patients are described in Table 1. They were female in 52% of cases, with a median age of 37 years. Compared to patients with encephalitis, those with meningitis were significantly younger (median age of 36 vs 51 years, *P* = .0048; Figure 2) and less often living with human immunodeficiency virus (HIV) (3.2% vs 58%, *P* = .0001). Other immunosuppressive conditions were not significantly different between the 2 groups. Patients with encephalitis had a longer latency to presentation (8 vs 5 days, *P* = .0234).

**Table 1.** Comparison of Baseline Characteristics of Patients With Central Nervous System Varicella Zoster Virus Infection by Univariate Analysis

Demographics	Total	Meningitis	Encephalitis	*P* Value
	(N = 135)	(n = 123)	(n = 12)	
Sex, female	70/135 (52)	63/123 (51)	7/12 (58)	.7678
Age, y, median (IQR)	37 (27–56)	36 (27–54)	51 (45–71)	.**0048**
BMI ≥30 kg/m^2^ (n = 116)	19/114 (17)	17/102 (17)	2/12 (17)	.9999
Immunosuppression				
Diabetes mellitus	5/135 (3.6)	3/123 (2.4)	2/12 (17)	.0609
HIV	11/135 (8)	4/123 (3.2)	7/12 (58)	.**0001**
Other immunosuppressive conditions^a^	11/135 (8.1)	11/123 (8.9)	0/12 (0)	.5997
Clinical presentation				
Duration of symptoms, d, median (IQR)	5 (3–7)	5 (3–6)	8 (4–15)	.**0234**
Abnormal qSOFA score	6/135 (4.4)	2/123 (1.6)	4/12 (33)	.**0005**
GCS score <15	9/135 (6.6)	3/123 (2.4)	6/12 (50)	.**0001**
Confusion	9/135 (6.6)	3/123 (2.4)	6/12 (50)	.**0001**
Seizures	2/135 (1.5)	0/123 (0)	2/12 (17)	.**0021**
Headache	134/135 (98)	123/123 (100)	9/12 (75)	.**0005**
Fever	50/135 (37)	45/123 (37)	5/12 (42)	.7579
Neck stiffness	48/135 (36)	45/123 (37)	3/12 (25)	.5253
Nausea and/or vomiting	91/135 (67)	90/123 (73)	1/12 (8.3)	.**0001**
Photo/phonophobia	80/135 (59)	78/123 (63)	2/12 (17)	.**0034**
Aphasia	7/135 (5.1)	1/123 (0.8)	6/12 (50)	.**0001**
Motor deficiency	5/135 (3.6)	1/123 (0.8)	4/12 (33)	.**0002**
Sensory deficiency	5/135 (3.6)	2/123 (1.6)	3/12 (25)	.**0047**
Radiculitis	14/135 (10)	14/123 (11)	0/12 (0)	.6119
Cranial nerve palsy^b^	13/135 (10)	13/123 (10)	0/12 (0)	.6045
Herpes zoster^c^	56/135 (41)	53/123 (43)	3/12 (25)	.3586
Time of shingles onset compared to neurological signs, d, median (IQR)	+1 (−1 to 3)	+1 (−1.5 to 3)	+1.5 (0.75–2.25)	.8345
VZV pneumonitis	0/135 (0)	0/123 (0)	0/12 (0)	.9999

Data are presented as No. (%) unless otherwise indicated. *P* values in bold indicate statistically significant variables.

Abbreviations: BMI, body mass index; GCS, Glasgow Coma Scale; HIV, human immunodeficiency virus; IQR, interquartile range; qSOFA, quick Sequential Organ Failure Assessment; VZV, varicella zoster virus.

^a^Organ transplant (n = 1), solid cancer (n = 1), hematological cancer (n = 1), systemic autoimmune disease (n = 8), corticosteroids (n = 6), other immunosuppressants (n = 7).

^b^Cranial nerves affected: III (n = 2), V (n = 2), VII (n = 11), VIII (n = 6), IX (n = 2), X (n = 3), XI (n = 1).

^c^Location of shingles: facial (n = 9), Ramsay Hunt area (n = 6), cervical (n = 6), thoracic (n = 27), lumbar (n = 2), sacral (n = 5), disseminated (n = 1).

### Clinical Description of VZV Meningitis

All patients with VZV meningitis had unusual headaches, associated with nausea and/or vomiting in 73%, photo/phonophobia in 63%, neck stiffness in 37%, and fever in 37% of cases. Fourteen patients (11%) had focal peripheral neurological signs, related to cranial nerve involvement (n = 13) or plexopathy (n = 1). HZ was present in 43% of cases, with main location in thoracic dermatomes (n = 27) and median onset time of 1 day after meningeal symptoms appeared. No patient had VZV-related pneumonia and only 1 patient had VZV-related hepatitis.

### Diagnostic Workup for VZV Meningitis

Paraclinical features of patients with VZV meningitis are described in Table 2. Most of them did not have systemic inflammation. Lumbar puncture (LP) was performed on the day of admission for 83% of patients. CSF was mostly clear with a median pleocytosis of 272 cells/μL and a strong predominance of lymphocytes. The median CSF protein level was 1.1 g/L with extreme values of 0.3–3.1 g/L. CSF glucose was normal in 82% of cases.

**Table 2.** Comparison of Paraclinical Features and Management of Patients With Varicella Zoster Virus Central Nervous System Infection by Univariate Analysis

Feature	Meningitis	Encephalitis	*P* Value
	(n = 123)	(n = 12)	
Blood analysis			
WBC count, × 10^9^/L (reference range, 4–10)	6.7 (5.6–8.2)	6.8 (5.1–8.6)	.8542
Lymphocytes, × 10^9^/L (reference range, 1–4)	1.6 (1.1–2.1)	1.5 (0.8–2)	.4116
C-reactive protein, mg/L			
≤5	104/122 (85)	7/12 (58)	.**0314**
>5	18/122 (15)	5/12 (42)	
CSF analysis			
Time to LP, d	0 (0–0)	2 (1–5)	.**0001**
Macroscopic			
Clear	97/123 (78)	11/12 (92)	.4601
Slightly turbid	28/123 (22)	1/12 (8)	.4588
WBC count, cells/μL (reference range, <5)	272 (117–459)	33 (5–119)	.**0001**
Lymphocytes, %	95 (90–97)	96 (85–99)	.9663
Polymorphonuclear, %	1 (0–2)	1 (0–8.5)	.3724
Protein, g/L (reference range, <0.45)	1.1 (0.7–1.6)	0.8 (0.6–1)	.2015
Glucose, mM (reference range, 2.5–4.5)	2.9 (2.5–3.2)	3 (2.9–3.4)	.1607
Positive bacterial culture	0/123 (0)	0/12 (0)	.9999
Positive VZV PCR			
Triplex	49/50 (98)	10/10 (100)	
Multiplex	84/86 (98)	3/3 (100)	
Both	10/13 (77)	1/1 (100)	
Other viruses by PCR^a^	4/123 (3.3)	5/12 (42)	.**0002**
Brain imaging			
Pathological/performed			
Brain CT	0/98 (0)	6/12 (50)	.**0001**
Brain MRI	8/44 (18)^b^	8/11 (73)	.**0011**
Both CT and MRI	5/33 (15)	8/11 (73)	.**0008**
Cerebral vasculitis	0/109 (0)	6/12 (50)	.**0001**
Electroencephalography			
Pathological/performed	2/8 (25)^c^	1/5 (20)	.9999
Hospital care			
Hospitalization	91/123 (74)	12/12 (100)	.1221
Hospital LOS, d	6 (4–9)	19 (14–35)	
Admission to ICU	4/123 (3.3)^d^	3/12 (25)	.**0156**
Antiviral therapy			
Acyclovir ≥7 d	31/123 (25)	12/12 (100)	.**0001**
Duration, d	9 (7–10)		
Dosage, mg/kg/8 h	10 (10–15)		
Acyclovir <7 d	51/123 (41)	0/12 (0)	.**0035**
Duration, d	2 (2–4)		
With oral switch	43/51 (84)		
Oral duration, d	8 (7–10)		
Without oral relay	8/51 (16)		
Valacyclovir only	24/123 (20)	0/12 (0)	.1241
Duration, d	7 (7–10)		
Dosage, g/d	3 (3–3)		
No antiviral therapy	17/123 (14)	0/12 (0)	.3616
Other therapies			
Empiric antibiotics^e^	14/123 (11)	4/12 (33)	.0557
Corticosteroids	6/123 (4.9)	1/12 (8)	.4869

Data are expressed as No. (%) or median (interquartile range). *P* values in bold indicate statistically significant variables.

Abbreviations: CSF, cerebrospinal fluid; CT, computed tomography; ICU, intensive care unit; LOS, length of stay; LP, lumbar puncture; MRI, magnetic resonance imaging; PCR, polymerase chain reaction; VZV, varicella zoster virus; WBC, white blood cell.

^a^Other viruses found: Epstein-Barr virus (n = 1), enterovirus (n = 1), human herpesvirus 6 (n = 1), human immunodeficiency virus (n = 7), JC virus (n = 1).

^b^Hypersignal of cranial nerves (n = 7), labyrinthitis (n = 5), leptomeningitis (n = 1).

^c^Hypovoltage (n = 2).

^d^Massive pulmonary embolism (n = 1), hemofiltration for acute kidney injury (n = 1), 24-hour monitoring (n = 2).

^e^Penicillin (n = 13), cephalosporin (n = 10), carbapenem (n = 1), aminoglycoside (n = 3), anti-tuberculosis drugs (n = 1).

VZV diagnosis was made mainly by multiplex PCR. In patients with HIV (n = 11), HIV was concomitantly detectable in CSF in 64% of cases, highlighting the importance of comprehensive viral screening in immunocompromised patients. The bacterial culture was negative in all cases.

Brain imaging was performed in 109 patients with meningitis: 90% of them had a CT scan that was always normal and 40% of them had an MRI that showed specific abnormalities in 8 patients (18%), such as labyrinthitis or hypersignal of cranial nerves. On physical examination, all 8 patients had peripheral facial palsy, associated with another cranial nerve involvement in 88% of cases (mainly nerve VIII). None of the patients with meningitis had signs of vasculitis on brain imaging.

Compared to meningitis, patients with encephalitis more often had elevated C-reactive protein, LP was performed later and showed lower pleocytosis, and brain imaging was significantly more pathological, showing evidence of cerebral vasculitis in half of them.

### Management and Outcomes of Patients With VZV Meningitis

Among the 123 patients with VZV meningitis, 91 (74%) were hospitalized with a median length of stay of 6 days. Admission to intensive care unit was rare (4/123 [3%]).

Overall, 82 of 123 (67%) patients received IV acyclovir with a median dose of 10 mg/kg 3 times daily (Table 2). Among them, 31 patients received IV acyclovir for at least 7 days (median, 9 days) and 51 patients for <7 days (median, 2 days). Patients with shorter duration of acyclovir switched to oral valacyclovir in 84% of cases, for a median duration of 8 days. Twenty-four (20%) patients were treated with valacyclovir alone and 14% did not receive antiviral treatment.

In addition to antivirals, 11% of patients received empiric antibiotics for a median duration of 2 days and 4.9% received a short course of corticosteroids for cranial root involvement.

The outcome was favorable in most cases, with only 2 patients having a delta mRS of 2 points, and 3 deaths that were not directly related to VZV infection (Table 3). Both patients with neurological sequelae had radiculitis: cervical plexopathy with deficit of the upper limb and paralysis of the third cranial nerve. They had incomplete recovery at discharge and at the end of their follow-up despite initial treatment with IV acyclovir. The median length of follow-up was 45 weeks, considering patients for whom we had follow-up data. None of the patients relapsed during follow-up. Thirty-eight patients (31%) were considered lost to follow-up because they had never been readmitted to a public hospital in Paris, but none of them were registered in the public national death registry at the end of the study.

**Table 3.** Characteristics, Management, and Outcomes of Varicella Zoster Virus Meningitis According to Antiviral Regimen

Demographics	All VZV Meningitis	Acyclovir ≥7 d	Acyclovir <7 d	Valacyclovir Only	No Antivirals	*P* Value
	(n = 123)	(n = 31)	(n = 51)	(n = 24)	(n = 17)	
Age, y	36 (27–54)	53 (38–61)	30 (24–37)	33 (28–45)	36 (29–56)	.**0004**
mRS before admission	0 (0–0)	0 (0–0)	0 (0–0)	0 (0–0)	0 (0–0)	.13
Immunosuppression						
Immunosuppressive condition^a^	17/123 (14)	10/31 (32)	5/51 (10)	2/24 (8.3)	0/17 (0)	.**008**
Clinical presentation						
Cranial nerve involvement	13/123 (10)	9/31 (29)	3/51 (5.7)	1/24 (4.2)	0/17 (0)	.**004**
Herpes zoster	53/123 (43)	15/31 (48)	21/51 (41)	15/24 (63)	2/17 (12)	.**010**
Biological analysis						
CRP >5 mg/L	18/122 (15)	9/31 (29)	6/51 (12)	3/24 (13)	0/16 (0)	.**026**
Protein in CSF, g/L	1.1 (0.7–1.6)	1.07 (0.6–1.8)	0.95 (0.6–1.4)	0.98 (0.8–1.3)	1.44 (0.8–1.6)	.42
Brain imaging						
Brain MRI performed	44/123 (36)	22/31 (71)	13/51 (25)	5/24 (21)	4/17 (24)	.**0001**
Abnormalities on MRI	8/44 (18)	6/22 (27)	2/13 (15)	0/5 (0)	0/4 (0)	.59
Management						
Hospitalization	91/123 (74)	31/31 (100)	51/51 (100)	3/24 (13)	6/17 (35)	.**0001**
Hospital LOS, d	6 (4–9)	10 (8–14)	4 (3–6)	0 (0–0)	0 (0–2)	.**0001**
Outcomes						
Unfavorable outcome	5/123 (4)	2/31 (6.5)	3/51 (5.9)	0/24 (0)	0/17 (0)	.64
Death^b^	3/123 (2.4)	1/31 (3.2)	2/51 (3.9)	0/24 (0)	0/17 (0)	1.00
mRS delta ≥2	2/123 (1.6)	1/31 (3.2)	1/51 (1.9)	0/24 (0)	0/17 (0)	.86
Acute kidney injury^c^	15/123 (12)	7/31 (23)	8/51 (16)	0/24 (0)	0/17 (0)	.**015**
Follow-up						
Lost to follow-up	38/123 (31)	6/31 (19)	15/51 (29)	10/24 (42)	7 (41)	.24
Follow-up duration, wk						
All patients (n = 123)	9 (1–99)	22 (6–97)	7 (1–84)	1 (0–36)	13 (0–159)	.**038**
Only those with follow-up	(n = 85)	(n = 25)	(n = 36)	(n = 14)	(n = 10)	
	**45 (7–165)**	36 (7–207)	39 (7–145)	29 (7–152)	135 (47–190)	
Relapse during follow-up	0/85 (0)	0/31 (0)	0/51 (0)	0/24 (0)	0/17 (0)	

Data are expressed as No. (%) or median (interquartile range). *P* values in bold indicate statistically significant variables.

Abbreviations: CRP, C-reactive protein; CSF, cerebrospinal fluid; LOS, length of stay; MRI, magnetic resonance imaging; mRS, modified Rankin Scale; VZV, varicella zoster virus.

^a^Including primary immunodeficiency, diabetes mellitus, human immunodeficiency virus status, solid or hematological cancer, organ transplant, corticosteroids, and other immunosuppressants.

^b^Massive pulmonary embolism (n = 1), cardiac arrest due to swallowing disorder related to Parkinson’s disease (n = 1), urinary septic shock (n = 1).

^c^Defined by the Kidney Disease—Improving Global Outcomes score as an acute creatinine increase of 0.3 mg/dL.

### Characteristics and Outcomes of VZV Meningitis According to Antiviral Regimen

Regarding history and prior autonomy, there was no difference between the 4 treatment groups based on the Kruskal-Wallis or the Fisher exact test (Table 3), but patients who received long duration of IV acyclovir were significantly older (median age, 53 [interquartile range {IQR}, 38–61] years vs 30 [IQR, 24–37] years in those with short-duration acyclovir, 33 [IQR, 28–45] years in those who received valaciclovir, and 36 [IQR, 29–56] years in those with no antivirals; *P* = .0004) and more often immunosuppressed (32%, 10%, 8%, and 0%, respectively; *P* = .008).

Focusing on clinical presentation, patients with HZ were more often treated with valacyclovir, regardless of the location of shingles. Conversely, the patients with cranial radiculitis more often received IV acyclovir for a longer duration (9/13 [69%]) than short duration (3/13 [23%]) or valaciclovir (1/13 [8%]) (*P* = .004). Regarding paraclinical findings, there was no evidence that the level of CSF inflammation (WBC and proteins) differed between treatment groups. In multivariate analysis, only age, underlying immunosuppression, and cranial radiculitis appear to be predictive factors for longer IV therapy, based on the AIC (Table 4). Length of hospitalization increased with the duration of IV acyclovir (median, 4 [IQR, 3–6] days in short duration of acyclovir and up to 10 [IQR, 8–14] days in those with long duration of acyclovir; *P* < .0001). One-third of patients who did not receive antivirals were still briefly hospitalized for symptomatic treatment and monitoring. There was no significant difference in unfavorable outcome between the 4 treatment groups, with no relapse and no death or neurological sequelae in the valacyclovir and no antivirals groups. Of the 15 of 123 patients (12%) who developed acute kidney injury, all but 1 received IV hydration, and 1 required hemodialysis for 3 days. At discharge, 8 of 15 patients (53%) had still not recovered their baseline creatinine level. The proportion of acute kidney injury appeared to be related to the duration of acyclovir (*P* = .015), but after adjustment for age and underlying immunosuppression, this difference was no longer significant (*P* = .30).

**Table 4.** Predictive Factors for Long Versus Short Intravenous Treatment After Multivariate Analysis With Variable Selection Using Akaike Information Criterion

Factor	OR	(95% CI)	*P* Value
Age	0.97	(.94–1.00)	.02
Cranial radiculitis			
No	1.00	(.05–1.15)	.09
Yes	0.27		
Immunosuppressive condition			
No	1.00	(.08–1.06)	.07
Yes	0.30		

Abbreviations: CI, confidence interval; OR, odds ratio.

## DISCUSSION

To our knowledge, this is the largest cohort of microbiologically proven VZV meningitis. Through our emergency headache reference center and the routine performance of multiplex PCR in CSF, we have been able to identify an increasing number of VZV meningitis over the last 8 years. In this study, we found that VZV meningitis affects young and immunocompetent patients with a good neurological and general prognosis even in patients treated with oral therapy or no antivirals.

The main strength of our study was its stringent inclusion and exclusion criteria. First, we distinguished meningitis from meningoencephalitis, and included only patients whose VZV infection had been confirmed by PCR in CSF, unlike previous major works [15, 19]. Then, we excluded patients without signs of meningeal irritation as it is known that viral DNA can be detected in CSF of up to one-third of immunocompetent patients with HZ without neurological signs [26]. Indeed, almost half of the patients with HZ may have concomitant CSF inflammation without neurological complaints [26, 27]. We described the epidemiology of VZV meningitis compared to VZV encephalitis. Although the number of patients with encephalitis was low in our cohort, our data were consistent with the nationwide prospective cohort of the Danish Study Group of Infections of the Brain (DASGIB), which reported 92 cases of VZV encephalitis with a median age of 75 years and 39% of immunocompromised patients [28]. The preponderance of young immunocompetent patients in our meningitis group is also consistent with recent prospective observational cohort studies [19, 29] and raises the hypothesis of a different pathophysiology from that of HZ or encephalitis, with possible genetic and immunological factors that remain to be elucidated. Furthermore, during follow-up, no patients with meningitis, even those who did not receive antiviral treatment, progressed to encephalitis or cerebral vasculitis, reinforcing the idea of a different pathophysiology.

In our study, meningitis was associated with HZ in 43% of cases, but this frequency may be underestimated in these immunocompetent patients with a small extent and/or a hidden location of HZ (eg, ear concha). Interestingly, the rash could precede meningeal signs by several weeks or appear several days after (delay ranging from −21 to +10 days in our cohort). In addition, 10% of patients had associated radiculitis of 1 or more cranial roots. The frequency of this association is difficult to define, as Ramsay Hunt syndrome is often managed by otorhinolaryngologists, who do not systematically perform LP if meningeal signs are not in the foreground [30]. However, a Korean study describing CNS infections caused by VZV and HSV pointed out that cranial nerves were affected only in the VZV group [8]. Interestingly, the DASGIB showed that patients with Ramsay Hunt syndrome and concurrent VZV meningitis had less pronounced meningeal symptoms than VZV meningitis without cranial nerve palsy, but functional outcomes were similar, with sequelae often related to cranial neuropathies [31]. In the absence of typical HZ, diagnosis of VZV meningitis is based on the detection of viral DNA by PCR in CSF or intrathecal specific immunoglobulins. In France, this latter biological test is no longer routinely performed. However, a recent meta-analysis of the accuracy of the Biofire FilmArray ME panel diagnostic test showed very good results for VZV identification, with a sensitivity of 91.4% and a specificity of 99.8% [31]. Therefore, the number of missed diagnoses was expected to be low. CT scan (with contrast in 81% of cases) was always normal. Brain MRI (with contrast in 86% of cases) was more frequently performed in patients with meningitis treated for longer duration with acyclovir, possibly due to the higher proportion of cranial radiculitis in this group. It shows labyrinthitis and contrast of cranial root in patients with cranial palsies on physical examination. These results suggest that brain imaging is without clinical value and potentially harmful for young patients undergoing X-ray examinations if viral meningitis is obvious.

Regarding the outcomes, only 2 patients of our cohort had neurological sequelae resulting in radiculitis with incomplete recovery despite initial treatment with IV acyclovir. None of the patients died from unfavorable evolution of the meningitis. A French study attempted to determine prognostic factors for VZV neurologic complications by reporting 26 cases of meningitis, 22 cases of meningoencephalitis, and 15 cases of cranial radiculitis [17]. In this study, meningitis without other neurological involvement had a good prognosis, whereas radiculitis involved a higher risk of functional sequalae and meningoencephalitis had a higher risk of mortality, which is consistent with our results. One of the limitations of our study is that the evaluation of the mRS score was not carried out by independent assessors, which may constitute an ascertainment bias. In addition, this assessment criterion does not consider neuro-psychological sequelae, which can have an impact on patients’ quality of life after meningitis but can only be assessed prospectively [29].

The originality of our study was to describe therapeutic management of VZV meningitis without encephalitis in the absence of specific recommendations from IDSA [27]. In our cohort, 17 patients (14%) received no antivirals, whereas 24 patients (20%) were treated with oral valacyclovir alone, 51 patients (41%) with a short course of IV acyclovir with oral relay, and 31 patients (25%) with a long course of IV acyclovir. Our main finding was that outcome was good, independent of antiviral regimen. This result brings to mind the 2015 retrospective study on the management of HSV-1 and HSV-2 meningitis, which provided reassuring data for symptomatic treatment alone in immunocompetent patients [22]. A prospective English study published in 2018 emphasizes that patients with VZV meningitis are more often treated with antivirals in clinical practice than those with HSV meningitis (56% vs 33%, *P* = .026) [29]. More recently, a prospective Danish study on viral meningitis concluded that early antiviral treatment was not associated with an improved outcome in meningitis caused by HSV-2 or VZV, in either immunocompetent or immunosuppressed patients [19]. In our study, older or immunocompromised patients and/or those with associated radiculitis were preferentially treated with a long course of IV acyclovir. This selection bias prevents us from proposing to treat these patients with oral valacyclovir. Although pharmacokinetic studies have demonstrated the good bioavailability of valacyclovir (55% vs 25% for oral acyclovir) and its good CSF penetration (54%, comparable to meningeal diffusion of IV acyclovir) [32], the rapid virucidal activity of IV acyclovir could remain of interest in patients at higher risk of functional sequelae.

The main limitation of our study was the absence of prospective follow-up, which may constitute a potential bias in the analysis of outcomes. However, in Paris, patient medical records are computerized on a specific software shared by all public hospitals. Consequently, if a patient had been hospitalized in another hospital due to unfavorable evolution, we would have had access to this information. As we cannot exclude private medical consultations, we checked the absence of deaths in the national public death register for patients who did not have a medical visit after discharge. As no deaths were recorded, we assume that the absence of subsequent medical visits in the entire network of public assistance hospitals in Paris was in favor of a good outcome in these young patients without comorbidities.

In conclusion, our study seems to support the idea that VZV meningitis is a milder complication than other VZV-related CNS involvements and thus may benefit from a milder treatment.

While our data need to be confirmed by prospective cohorts, they provide reassuring information to consider future controlled trials comparing acyclovir to valacyclovir or no antivirals for the management of VZV meningitis without other neurological complication in immunocompetent patients.

## Note

***Potential conflicts of interest*.** All authors: No reported conflicts of interest.
